# Comparing video examinations with physical clinical examinations using finishing pigs with umbilical outpouchings as a model

**DOI:** 10.1186/s13028-023-00689-8

**Published:** 2023-06-24

**Authors:** Julie Melsted Birch, Ken Steen Pedersen

**Affiliations:** 1 Section for Production, Nutrition and Health, Department of Veterinary and Animal Sciences, Faculty of Health and Medical Sciences, University of Copenhagen, Groennegaardsvej 2, 1870 Frederiksberg C, Denmark; 2 Present Address: Section for Pathobiological Sciences, Department of Veterinary and Animal Sciences, Faculty of Health and Medical Sciences, University of Copenhagen, Ridebanevej 3, 1870 Frederiksberg C, Denmark; 3 Ø-Vet A/S, Køberupvej 33, 4700 Næstved, Denmark

**Keywords:** Agreement, Fitness for transport, Inter-observer agreement, Sus scrofa, Umbilical hernia, Veterinary telemedicine, Wound

## Abstract

**Background:**

Veterinary telemedicine has only been adopted to some degree. One aspect that needs to be evaluated is clinical examinations using video. The objective of this study was to evaluate agreement between a traditional physical clinical examination and a clinical examination using recorded video using finishing pigs with umbilical outpouchings (umbilical hernias, cysts, and abscesses) as the study unit. A total of 102 finisher pigs with umbilical outpouchings were clinically examined and recorded on video. Four experienced pig veterinarians were allowed to examine each pig for approximately 10 min and were individually asked to fill out a predesigned clinical record. Approximately 1 month after the physical examinations, the veterinarians individually reexamined all 102 pigs in a blinded manner, utilizing the video recordings and filling in a predesigned clinical record.

**Results:**

For all measurements using a ruler, a high Pearson correlation coefficient was observed between physical and video examinations (range 0.69–0.95). In comparison, the visual bodyweight estimation had a lower Pearson correlation coefficient (range 0.57–0.64). Substantial to almost perfect agreement was observed between the physical and video examinations for abnormal weight distribution on any leg, restricted gait movements, lameness, signs of pain, fitness for transportation, presence of wounds, and categorization of the number of wounds > 4 cm^2^ on the umbilical outpouching (mean Kappa range 0.67–0.87). Fair agreement was observed for the presence of perineal soiling, ear wounds, pendulation of umbilical outpouching, umbilical outpouching touching the legs, skin not movable over the umbilical outpouching, and umbilical outpouching wound characteristics: type, presence of crusts, active bleeding, thick wound edges, connective tissue (mean Kappa range 0.21–0.40). Slight agreement was observed for umbilical outpouching characteristics: shape, macroscopic vascularization of the skin covering the outpouching, and the presence of scars, excoriations, and fistulas (mean Kappa range 0.10–0.20). Poor agreement was observed for the presence of granulation tissue (mean Kappa =  − 0.05).

**Conclusions:**

The agreement between a physical clinical examination and a clinical examination using recorded video of the same pig varies from poor to almost perfect, depending on the clinical sign and the executing veterinarian.

**Supplementary Information:**

The online version contains supplementary material available at 10.1186/s13028-023-00689-8.

## Background

The developments in information and communication technologies (ICT) have rapidly been adopted in several human medical disciplines. Tele-radiology, -dermatology, -pathology and -psychology were the first to evolve. However, today “telemedicine” or “telehealth” are used in a broader consumer facing approach as health services are delivered by professionals such as nurses, pharmacists, etc. [1]. The exchange of information is either synchronous (e.g., live video consultation or live electrocardiogram) or asynchronous (store-and-forward) when, e.g., video, images, or bio-signals are recorded and exchanged at a convenient time. Today, human telemedicine extends to tele-trauma care, -cardiology, -ophthalmology, -surgery, remote patient monitoring of chronic diseases using digital wearables, and many more disciplines. These new modalities have increased the request for evidence-based studies, and practice guidelines are available in human medicine [2].

Veterinary telemedicine has only been adopted to some degree, including phone consultations. The information can be exchanged between a veterinarian and a client (a farmer or pet owner) focusing on a single animal or a group of animals. Recently, some Danish companion animal clinics started offering teleconsultation out of hours as an additional triage service, and one pig practice started offering videoconsultation for the evaluation of sows in sick-pens in addition to the ordinary farm-visits. Literature in veterinary telemedicine often describes its application in the information exchange between the general practitioner and the specialist in order to improve diagnostics in companion animal medicine [3]. In addition, the effects on the veterinarian-client-patient-relationship (VCPR) and the perception of teleconsultation by the pet owner and the veterinarian have been in focus [4].

In a veterinary context, the technology has some obvious applications in regards to farmed animals: It would save the veterinarian's traveling time and therefore potentially optimize the veterinarian-client contacts. The extended internet networks and mobile devices facilitate access to veterinary knowledge in rural areas. Making decisions would be easier with a veterinarian online, who could assist unexperienced farmers or staff and potentially alleviate “care gaps” or make follow-ups easier. One potential application of telemedicine is clinical examinations and making a diagnosis on farmed animals. A veterinarian working remotely might use live or recorded videos to assess an animal’s fitness for transport or make choices about whether to administer antibiotics. Implementation of teleservices in veterinary medicine has raised discussion among professionals. Veterinary telemedicine is often compared to pediatrics because the patients (animals) are not able to communicate their feelings or complaints [3]. Legal and ethical concerns, including effectiveness in predicting health status, the risk of misdiagnosis, information security, legal responsibility, medical prescription, the need for an established VCPR, and acceptance from the national regulatory authorities, have been discussed [4]. These concerns are much the same as the ones that continuously need to be addressed in human telemedicine [1]. The pros and cons of telemedicine in veterinary clinical practice need to be uncovered, and both technical and scientific aspects need to be addressed. One aspect that needs to be evaluated is the aforementioned clinical examinations using live or recorded video of animals. To our knowledge, no studies have evaluated clinical examinations of animals using video. The utilization of such video examinations and the agreement to a traditional physical clinical examination could be different between various clinical signs, but this aspect will be uncovered as studies are performed and reported.

Umbilical outpouchings are a clinical term for umbilical hernias, cysts, and abscesses in the umbilical region of pigs, and this condition is highly prevalent in Danish pig production [5]. Umbilical outpouchings hold a welfare risk for the individual pig because of the risk of developing ulcers and other complications. Often, these pigs are euthanized, contributing to an increased mortality rate. Therefore, video examination of pigs with umbilical outpouchings would be very relevant in the pig industry and is one of many potential relevant conditions and clinical signs when considering the implementation of telemedicine in large animal practice.

The objective of this study was to evaluate agreement between a traditional physical clinical examination and a clinical examination using recorded video, using finishing pigs with umbilical outpouchings as the study unit.

## Methods

### Study design

During 4 days, 102 finisher pigs (61 females, 40 castrated males, and 1 intact male) with umbilical outpouchings were selected. A wound on the umbilical outpouching was selected by the leader of the experiment for 49 pigs for separate assessment. All the pigs originated from two herds owned by the same Danish farmer. The pigs were housed in sick pens together with other pigs with different kinds of umbilical outpouchings. Each pig was individually ear-tagged the day before the experiment was carried out. On the day of the experiment, one by one, each pig was separated in a smaller compartment, recorded on video, and examined by 4 experienced (8–20 years in pig practice) pig veterinarians (Vet 1–4). After the general examination was performed, the outpouchings (and the wounds) were examined. The video recordings from each pig were edited and blinded with new pig ID numbers (Video Case 1–102). Approximately 1 month after the physical examinations, the veterinarians individually reexamined all pigs in a blinded manner, utilizing the video recordings and filling in a predesigned clinical record. In this manner, two sets of clinical assessments were generated, a physical (EX_PHYS) and a video-based (EX_VIDEO), for each pig by all four veterinarians.

### Clinical record

No definition of any clinical sign and condition was provided, and no calibration between veterinarians was performed. The clinical record was designed as a pre-printed paper version of a questionnaire with closed questions using dichotomous or polytomous answer possibilities for categorical outcomes. Questions demanding quantitative outcomes were designed as open questions with a predefined unit on a continuous scale. The record was divided into a general part, a part regarding the outpouching, a part regarding the selected wound on the outpouching, and a part with a final conclusion. Each clinical record was provided with the veterinarian's initials and pig ID, either the ear tag number (EX_PHYS) or the video case number (EX_VIDEO). Tables 1 and 2, as well as the Additional files 2, 3, 4 and 5, contain all clinical signs and conditions included in the clinical records.

**Table 1:** Agreement between clinical examinations performed physically in the stable or by recorded video

Clinical measurement	Veterinarian 1		Veterinarian 2		Veterinarian 3		Veterinarian 4		Mean across veterinarians
	Difference (SD)*	Pearsons	Difference (SD)*	Pearsons	Difference (SD)*	Pearsons	Difference (SD)*	Pearsons	Pearsons (SD)
Weight in kg	2.2 (17.1)	0.64	0.34 (14.8)	0.62	− 5.8 (12.6)	0.57	− 6.7 (13.9)	0.57	0.60 (0.04)
Distance of outpouching from abdominal wall in cm	3.0 (2.9)	0.77	− 0.4 (2.0)	0.82	(a)	(a)	1.0 (2.4)	0.79	0.79 (0.03)
Width of outpouching in cm	0.9 (2.2)	0.87	0.3 (2.2)	0.78	2.1 (2.3)	0.81	0.5 (2.1)	0.80	0.82 (0.04)
Distance from outpouching to floor in cm	− 0.4 (3.1)	0.69	− 1.5 (2.4)	0.81	− 1.5 (2.6)	0.78	− 0.6 (2.2)	0.84	0.78 (0.06)
Wound length in cm	− 0.1 (0.8)	0.91	0.0 (0.7)	0.95	0.2 (0.9)	0.92	0.0 (0.7)	0.92	0.93 (0.02)
Wound width in cm	− 0.3 (1.0)	0.86	− 0.1 (0.6)	0.88	0.0 (0.7)	0.80	− 0.2 (0.5)	0.95	0.87 (0.06)
Wound length + wound width	− 0.4 (1.3)	0.92	− 0.1 (1.0)	0.95	0.3 (1.1)	0.93	− 0.2 (1.1)	0.94	0.94 (0.01)

The table displays the examined continuous measured clinical signs for 102 finishing pigs with umbilical outpouchings

(a) missing value because Veterinarian 3 misinterpreted the question

*Difference: measurement at physical clinical examination minus measurement at examination performed by recorded video. SD = Standard deviation. Pearsons = Pearson’s correlation coefficient

**Table 2:** Agreement between clinical examinations performed physically in the stable or by recorded video

	Veterinarian 1		Veterinarian 2		Veterianarian 3		Veterinarian 4		Mean^b^
	Agreement	Kappa (95% con)^a^	Agreement	Kappa (95% con)^a^	Agreement	Kappa (95% con)^a^	Agreement	Kappa (95% con)^a^	Kappa (SD)
*General examination*									
Abnormal weight distribution on one or more legs (yes/no)	0.99 (101/102)	0.80 (0.40; 1.00)	0.99 (101/102)	0.80 (0.40; 1.00)	0.99 (101/102)	0.80 (0.40; 1.00)	0.99 (101/102)	0.80 (0.40; 1.00)	0.80 (0.0)
Restricted gait movements (yes/no)	1.00 (102/102)	1.00 (1.0; 1.0)	0.96 (98/102)	0.65 (0.33; 0.97)	0.99 (101/102)	0.80 (0.40; 1.00)	0.98 (100/102)	0.74 (0.40; 1.00)	0.80 (0.15)
Lame (pig level) (yes/no)	0.99 (101/102)	0.8 (0.40; 1.00)	0.99 (101/102)	0.88 (0.66; 1.00)	0.98 (100/102)	0.66 (0.22; 1.00)	1.00 (102/102)	1.00 (1.00; 1.00)	0.84 (0.14)
Spontaneous signs of pain (yes/no)	1.00 (102/102)	1.00 (1.00; 1.00)	0.97 (96/99)	0.65 (0.28; 1.00)	0.99 (97/98)	–	0.98 (100/102)	0.49 (− 0.11; 1.00)	0.71 (0.26)
*Umbilical outpouching*									
Consistence (firm, soft)	0.91 (93/102)	0.62 (0.39; 0.84)	0.97 (99/102)	0.81 (0.60; 1.00)	0.80 (82/102)	0.22 (0.02; 0.43)	0.88 (89/101)	0.27 (− 0.02; 0.57)	0.48 (0.28)
Reducibility (yes/no)	0.88 (89/101)	0.60 (0.40; 0.80)	0.88 (89/101)	0.45 (0.19; 0.70)	0.70 (71/102)	0.28 (0.13; 0.43)	0.70 (69/98)	0.33 (0.16; 0.50)	0.42 (0.14)
Wounds present (yes/no)	0.94 (96/102)	0.88 (0.79; 0.97)	0.89 (90/101)	0.74 (0.61; 0.87)	0.94 (96/102)	0.88 (0.79; 0.97)	0.98 (99/101)	0.96 (0.91; 1.00)	0.87 (0.09)
Number of wounds > 4 cm^2^	0.88 (36/41)	0.76 (0.56; 0.95)	0.91 (30/33)	0.82 (0.62; 1.00)	0.86 (37/43)	0.71 (0.49; 0.92)	0.96 (43/45)	0.91 (0.79; 1.00)	0.80 (0.09)
*Specific wound examination*									
Location (bottom/side)	0.92 (45/49)	0.31 (− 0.16; 0.78)	0.85 (40/47)	0.29 (− 0.10; 0.67)	0.94 (46/49)	0.64 (0.19; 1.00)	0.85 (40/47)	0.39 (0.04; 0.74)	0.41 (0.16)
Necrosis present (yes/no)	0.94 (46/49)	0.63 (0.25; 1.00)	0.67 (33/49)	–	0.71 (35/49)	0.44 (0.21; 0.68)	0.94 (46/49)	0.37 (− 0.19; 0.93)	0.48 (0.13)
Severe wound (yes/no)	0.76 (37/49)	0.50 (0.26; 0.75)	0.84 (41/49)	0.61 (0.37; 0.84)	0.82 (40/49)	0.57 (0.33; 0.81)	0.80 (39/49)	0.39 (0.09; 0.68)	0.52 (0.10)
"Open" wound (yes/no)	0.90 (44/49)	0.26 (− 0.15; 0.67)	0.73 (36/49)	0.48 (0.25; 0.71)	0.90 (44/49)	0.26 (− 0.15; 0.67)	1.00 (49/49)	1.00 (1.00; 1.00)	0.50 (0.35)
Prognosis (guarded + poor)/good))	0.86 (42/49)	0.61 (0.35; 0.86)	0.76 (37/49)	0.19 (− 0.04; 0.43)	0.86 (42/49)	0.68 (0.47; 0.90)	0.71 (35/49)	0.24 (0.001; 0.49)	0.43 (0.25)
*Conclusion*									
Fit for transport (yes/with precautionary measures/no)	0.83 (85/102)	0.76 (0.65; 0.87)	0.69 (70/102)	0.54 (0.40; 0.67)	0.68 (69/102)	0.59 (0.47; 0.72)	0.88 (90/102)	0.79 (0.68; 0.90)	0.67 (0.12)

The table displays the clinical signs recorded as categorical variables and only those clinical signs where the mean Cohen’s Kappa value across the four Veterinarians was above 0.4 (n = 102 finishing pigs with umbilical outpouchings)

^a^Cohen’s Kappa value with 95% confidence interval limits

^b^Mean Cohen’s Kappa value across the four Veterinarians, SD = standard deviation

### Clinical physical examinations

All four veterinarians examined each pig in a separate compartment for about 10 min, and they were not allowed to discuss their findings with each other during any part of the physical clinical examinations. Each veterinarian was equipped with a 30 cm ruler and pre-printed clinical records to be completed. The pigs were not fixated, and the veterinarians were allowed to touch, palpate, and attempt to reduce the outpouching into the abdomen. Troubled pigs were managed by holding them in place with a driving board. If a wound was selected by the leader of the experiment, a dedicated part of the record was filled in before the final conclusion was stated. The examination stopped when all veterinarians expressed that they had finished filling out the records.

### Clinical video examinations

The video sequences were obtained from a Samsung Galaxy S9 + 64 GB (Samsung, Suwon-si, South Korea) smart phone camera mounted on a DJI Osmo Mobile 4 magnetic stabilizer (DJI, Nanshan, Shenzhen, China). The footage was obtained in a 16:9 format with 30 frames per second and stored as MP4 files. The flash light was set in automatic mode. However, when filming was performed underneath the pig, the flash light was applied.

The video filming of the pigs was conducted immediately before the physical examination by the two leaders of the experiment and in the absence of the veterinarians. One was holding the stabilizer with the camera, and the other person presented the pig in front of the camera. First, the ear tag was shot in close-up for identification of the pig. The pig was shot from the right and left sides, from behind, and from the front. The pig was urged to walk and turn around in order to present its movements. The face was filmed, and the outpouching was shot in close-up from both sides at a perpendicular camera angle. The person presenting the pig held a ruler in front of the outpouching for the size of the outpouching to be readable. Likewise, the ruler was held so that the distance from the abdominal wall to the deepest point of the outpouching and the distance from the outpouching to the floor were readable. The outpouching was lifted out in order to present the bottom of the outpouching to the camera. In addition, the outpouching was palpated and urged to be reduced into the abdomen by the presenting person. Any wounds were filmed in close-up, and a ruler was held in front of the wound to assess the size of the wound. Sequences between 3 and 7 min were recorded for each case, depending on the willingness of the pig.

The video sequences were edited using the Adope Premium Pro software (Adope, San Jose, CA, USA). A random video case number between 1 and 102 was added, and junk sequences were deleted. The ear tags were masked with the Gaussian blur effect. After the general presentation of the pig and the outpouching, a still frame was added with an instruction to stop the video and fill in the clinical record. If no wound was selected, the spectator was then asked to skip the selected wound section and complete the conclusion section. If a wound was selected, a dynamic red circle around the wound was added by using the mask and ramp features. Thereafter, the spectator was asked to fill in the wound section and complete the clinical record. The edited video sequences were between 1.48 and 3.43 min long, and the sound was muted throughout the whole sequence in order to mask the conversation between the scientific team members during the video recording. The edited sequences were exported in H.264 format and stored as MP4 files (see an example of a video case in Additional file 1).

The video cases were assembled in four folders enclosing 25–27 video cases, which were made accessible one by one from an internet link at weekly intervals. All four veterinarians were then instructed to conduct video-based clinical examinations, filling out the same clinical record questionnaire as the physical examinations. The veterinarians were allowed to revisit the whole or parts of the video sequences if necessary. In the case of missing data from the video examination, the veterinarians filled in the missing questions in a second round of data collection. For this purpose, a clinical record with the missing questions to be answered in addition to the relevant video case sequences was provided.

### Statistics

The collected data were entered into MS Excel (Microsoft, Redmond, WA, USA). The coding and distribution were checked for each variable before importation into SAS Enterprise Guide 7.1 (SAS Institute Inc., Cary, NC, USA). For all the continuous measured clinical signs, Pearson correlation coefficients between EX_PHYS and EX_VIDEO were performed using the proc corr command. The mean Pearson correlation coefficient for veterinarians 1–4 were calculated across the continuous measured clinical signs. For categorical variables, comparison between the EX_PHYS and EX_VIDEO was done by estimating simple agreement and Cohen’s Kappa values (Kappa values) by using the exact agree statement in the proc freq procedure. Pairs of observations (EX_PHYS and EX_VIDEO) where the EX_PHYS outcome was missing were deleted before any agreement analysis. Calculations of mean Kappa values were performed for each clinical sign across the four veterinarians and for each of the four veterinarians across the clinical signs. Kappa values were interpreted as < 0 indicating poor agreement, 0–0.20 as slight agreement, 0.21–0.40 as fair agreement, 0.41–0.60 as moderate agreement, 0.61–0.80 as substantial agreement, and 0.81–1 as almost perfect agreement [6].

## Results

Summary prevalence tables of the clinical signs recorded from the physical and video examinations are found in the Additional files 2, 3, 4 and 5. Most clinical signs had a low prevalence. From the physical examinations, clinical registrations were missing at random, so agreement analysis could not be performed for all pairs of clinical observations for all four veterinarians.

The results from weight estimation and measurements using the ruler are shown in Table 1. For all measurements using a ruler, a high Pearson correlation was observed between physical and video examinations (range 0.69–0.95). In comparison, the visual weight estimation had a lower Pearson correlation coefficient (range 0.57–0.64). For all the continuous measured clinical signs, the mean Pearson correlation coefficient for veterinarians 1–4 were 0.81, 0.83, 0.80, and 0.83, respectively. Numbers for agreement and Kappa values between physical and video examinations are shown in Table 2 for clinical signs with mean Kappa values above 0.4 across the four veterinarians. The interpretation of Kappa values according to [6] was as follows: Substantial to almost perfect agreement was found for abnormal weight distribution on any leg, restricted gait movements, lameness, signs of pain, the presence of wounds, the categorization of the number of wounds > 4 cm^2^, and the pig’s fitness for transportation (mean Kappa range 0.67–0.87). The presence of perineal soiling, ear wounds, pendulation of the umbilical outpouching, touching of the legs, the skin over the outpouching was not movable, wound type, the presence of crusts, active bleeding, thick wound edges, and connective tissue had a fair agreement (mean Kappa range 0.21–0.40). The agreement between the physical and video examinations of the umbilical outpouching's shape, macroscopic vascularization of the skin covering the outpouching, and the presence of scars, excoriations, and fistulas was slight (mean Kappa range 0.10–0.20), while the agreement of the presence of granulation tissue had a poor agreement (mean Kappa =  − 0.05). All four veterinarians concluded that fewer pigs were fit for transportation based on video examinations compared to physical examinations.

For those clinical signs in Table 2 with mean Kappa values above 0.4 across the four veterinarians, the mean Kappa value across the clinical signs: abnormal weight, distribution on one or more legs, restricted gait movements, lame (pig level), spontaneous signs of pain, umbilical outpouching consistence, umbilical outpouching reducibility, wounds present on umbilical outpouching, and fitness for transport was calculated for veterinarians 1–4 to be, 0.81 (SD = 0.14), 0.69 (SD = 0.14), 0.60 (SD = 0.24), and 0.67 (SD = 0.27), respectively.

## Discussion

Approximately half of the mean Kappa values between the physical and video examinations were found to have moderate, substantial, or almost perfect agreement, the other half had agreements ranging from slight to fair, and one clinical sign had a poor agreement between the physical and video clinical examinations. We found a large variation in the agreement among the clinical signs between the physical and video examinations. It was also shown that there was a difference between the veterinarians, where veterinarian 1 performed best and had the highest mean Kappa value and the smallest variation compared to the other veterinarians. However, this variation was not evident for the continuous outcomes, where measurements using a ruler had high Pearson correlations for all four veterinarians. It is worth noting that there seemed to be a tendency toward assessing animals as sick instead of normal based on the video. For example, this was the case with transport suitability, and more wounds were also assessed as “open” or “severe” during the video examinations compared to the physical examinations. This could possibly be a sign that the veterinarian has deemed it more difficult from the video and has therefore tended to assess something as being abnormal instead of normal. Alternatively, the possibility to pause the video during examination and the fact that the wounds were presented as magnified and better enlightened in the close-up video material could have disclosed the true status of the wounds.

Overall, the assessment of wounds seemed to be one of the things that was difficult for many of the veterinarians, as it was subject to more variation than many of the other clinical signs. A training of the veterinarians in advance of the study, including guidelines on how to assess the wounds according to the clinical record, would probably have resulted in less variation. Further, the terms “severe” and “open” were used for the classification of wounds, as these are included in the European Union legislation on fitness for transportation. However, these terms were not defined and were therefore assessed rather subjectively by each veterinarian.

Finally, the veterinarians were asked in respect of each animal if it was assessed to be fit for transportation under normal conditions or under special consideration for extra comfort. There was clearly a very different view of these terms among the veterinarians. Interestingly, the decision based on the video and physical examination was more consistent for each veterinarian than the decision amongst veterinarians. The study confirms results from previous studies that showed variation between veterinarians when performing clinical examinations [7, 8].

Agreement for many of the clinical signs could not be assessed because of low or no prevalence. This was expected because the pigs chosen for this study all had umbilical outpouchings, while the occurrence of other clinical signs was low. If the pigs in question had more variation in other clinical signs, the study could also have been used to assess the possibilities of utilizing video for other conditions than umbilical outpouchings. However, the pigs were selected with the exact purpose of representing large finisher pigs with umbilical outpouchings, which in many cases must be assessed for their transport suitability. In addition, we chose wounds on the umbilical outpouching, which was used as a model for evaluation of wounds irrespective of the location on the animal. In this way, the pigs represented finisher pigs with umbilical outpouchings well, as various sizes, shapes, and wounds were represented among the selected animals.

In this study, each veterinarian only performed one physical and video examination for each pig. It would have improved the strength of the study if each veterinarian could have performed repeated examinations of each pig, both physically and on video. This would have allowed for the estimation of intra-observer agreement for each veterinarian. However, this was not possible for practical reasons.

With regard to the purely practical circumstances during the video recording, the conditions were sufficient and very standardized on the farms used for examination. Furthermore, the video quality appeared adequate, and the use of the light source from the mobile phone provided a good light setting in respect of the things that needed to be clinically assessed. Regarding the implementation of clinical video examinations, we recommend custom-made pilot studies to be conducted. Specific clinical signs and the study group must be carefully selected with enough variation in order to evaluate the agreement. The use of rulers and other objective tools should be included whenever possible. In our view, when using recorded video, it is completely central to either use a predesigned clinical record, such as in this study, or alternatively use synchronous live video, making it possible for the observer to direct the person video recording, thereby getting further visualization of specific parts that the observer wants to examine further. We recommend that the presentation of each animal follow a set of criteria that must be fulfilled. The use of cameras and lights should also be standardized. However, a good smartphone camera with a magnetic stabilizer to minimize vibrations can be used in many cases. Finally, we recommend that training of the observers to be conducted in order to decrease the inter- and intra-observer variation.

We suggest that employing video recordings in clinical practice has a lot of potential in the future, provided that all of these conditions are met. It not only offers veterinary aid in remote places, but it also gives the working veterinarian more freedom during a typical workday because some animals can be examined remotely. The use of telemedicine in veterinary medicine has increased and is already applied in different situations [9]. However, there is a lack of studies comparing traditional physical clinical examinations with examinations performed by telemedicine. Furthermore, studies are needed to evaluate the safety and efficacy of telemedicine compared to traditional consultations. One study reported low prescribing rates (including antimicrobials), treatments were efficacious, and no harm was done by prescribing remotely via a veterinary video consult app [10]. Therefore, similar to our study, video consultations show promising results. However, this study also displays disagreements on conclusions related to important clinical decisions like fitness for transport (disagreement for 12–32% of pigs), severe wound (disagreement for 16–24% of pigs), and open wound (disagreement for 0–27% of pigs). Therefore, to prevent compromised animal welfare, we strongly advise more studies to be conducted before clinical examination using video can be applied for diagnosing new cases of disease and assessing fitness for transport.

## Conclusions

The agreement between a traditional physical clinical examination and a clinical examination using recorded videos varies from poor to almost perfect agreement depending on the type of clinical sign and the executing veterinarian. This highlights the fact that video-examination may be a valid method for some conditions and clinical signs but not for others. Before clinical video-examinations can be used for the assessment of fitness for transport of finishing pigs with umbilical outpouchings, we strongly recommend further research be done to prevent compromised animal welfare.

## Supplementary Information

**Additional file 1**: Video showing an example of a video case.

**Additional file 2**: Occurrence of clinical signs recorded during a traditional physical clinical examination in the stable (Physical) and a clinical examination of the same pigs (n = 102 pigs) performed by watching recorded video approximately 1 month after the physical examination (Video). The pigs all had umbilical outpouchings and were selected from two herds. Video recording of the individual pigs was made immediately before the physical examination was performed. All pigs were examined both physically and using video by the same four experienced pig veterinarians.

**Additional file 3**: Clinical examination results of umbilical outpouchings in finishing pigs (n = 102 pigs). The umbilical outpouchings were clinically examined during a traditional physical clinical examination in the stable (Physical) and a clinical examination of the same pigs performed by watching recorded video approximately 1 month after the physical examination (Video). The pigs all had umbilical outpouchings and were selected from two herds. Video recording of the individual pigs was made immediately before the physical examination was performed. All pigs were examined both physically and using video by the same four experienced pig veterinarians.

**Additional file 4**: Clinical examination results of wounds (n = 49 wounds) located on umbilical outpouchings in 49 different finishing pigs. The wounds were clinically examined during a traditional physical clinical examination in the stable (Physical) and a clinical examination of the same wounds performed by watching recorded video approximately 1 month after the physical examination (Video). The pigs all had umbilical outpouchings and were selected from two herds. Video recording of the individual pigs was made immediately before the physical examination was performed. All pigs were examined both physically and using video by the same four experienced pig veterinarians.

**Additional file 5**: Fitness for transport evaluation of 102 finishing pigs performed by four experienced pig veterinarians. All pigs were clinically examined during a traditional physical clinical examination in the stable (Physical) and a clinical examination of the same pigs was performed by watching recorded video approximately 1 month after the physical examination (Video). The pigs all had umbilical outpouchings and were selected from two herds. Video recording of the individual pigs was made immediately before the physical examination was performed. All pigs were examined both physically and using video by the same four experienced pig veterinarians.

## Data Availability

The datasets used and/or analyzed during the current study are available from the corresponding author on reasonable request.
