# Supplementary material for: Comparing video examinations with physical clinical examinations using finishing pigs with umbilical outpouchings as a model
Source: Acta Vet Scand. 2023 Jun 24;65:26. doi: 10.1186/s13028-023-00689-8 (PMC10290328; doi:10.1186/s13028-023-00689-8)
Supplement: Supplementary file 2 — Additional file 2: Occurrence of clinical signs recorded during a traditional physical clinical examination in the stable (Physical) and a clinical examination of the same pigs (n = 102 pigs) performed by watching recorded video approximately 1 month after the physical examination (Video). The pigs all had umbilical outpouchings and were selected from two herds. Video recording of the individual pigs was made immediately before the physical examination was performed. All pigs were examined both physically and using video by the same four experienced pig veterinarians. [file 13028_2023_689_MOESM2_ESM.docx]

**Additional file 2.** Occurrence of clinical signs recorded during a traditional physical clinical examination in the stable (Physical) and a clinical examination of the same pigs (n=102 pigs) performed by watching recorded video approximately one month after the physical examination (Video). The pigs all had umbilical outpouchings and were selected from two herds. Video recording of the individual pigs was made immediately before the physical examination was performed. All pigs were examined both physically and using video by the same four experienced pig veterinarians.

|  | | |  |  |  |  |  |  |
| --- | --- | --- | --- | --- | --- | --- | --- | --- |
|  | Veterinarian 1 | | Veterinarian 2 | | Veterinarian 3 | | Veterinarian 4 | |
|  | Physical | Video | Physical | Video | Physical | Video | Physical | Video |
| Visual estimation of body weight:  kg (Standard deviation) | 97.6 (21.9) | 95.4 (17.9) | 82.7 (17.2) | 82.4 (16.6) | 96.8 (15.3) | 102.6 (8.8) | 78.8 (16.3) | 85.5 (13.1) |
| Interest in surroundings (normal/abnormal) | 0 % (0/102) | 0 % (0/102) | 0 % (0/102) | 0 % (0/102) | 0 % (0/102) | 0 % (0/102) | 0 % (0/102) | 0 % (0/102) |
| Activity level: Inactive | 0 % (0/102) | 0 % (0/102) | 1 % (1/102) | 1 % (1/102) | 1 % (1/99) | 0% (0/102) | 2 % (2/102) | 3 % (3/102) |
| Normal | 100 % (102/102) | 100 % (102/102) | 99 % (101/102) | 99 % (101/102) | 97 % (96/99) | 100 % (102/102) | 98 % (100/102) | 97 % (99/102) |
| Hyperactive (yes/no) | 0 % (0/102) | 0 % (0/102) | 0 % (0/102) | 0 % (0/102) | 2 % (2/99) | 0% (0/102) | 0 % (0/102) | 0 % (0/102) |
| Weight distribution (normal/abnormal) | 2 % (2/102) | 3 % (3/102) | 2 % (2/102) | 3 % (3/102) | 3 % (3/102) | 2 % (2/102) | 2 % (2/102) | 3 % (3/102) |
| Head position (normal/abnormal) | 0 % (0/102) | 0 % (0/102) | 1 % (1/102) | 2 % (2/102) | 1 % (1/102) | 0 % (0/102) | 0 % (0/102) | 0 % (0/102) |
| Gait (restricted) (yes/no) | 3 % (3/102) | 3 % (3/102) | 7 % (7/102) | 5 % (5/102) | 3 % (3/102) | 2 % (2/102) | 3 % (3/102) | 5 % (5/102) |
| Lame pig (any leg) (yes/no) | 2 % (2/102) | 3 % (3/102) | 5 % (5/102) | 4 % (4/102) | 4 % (4/102) | 2 % (2/102) | 3 % (3/102) | 3 % (3/102) |
| Body condition: Slim | 0% (0/102) | 0% (0/102) | 0% (0/102) | 0% (0/102) | 0% (0/102) | 0% (0/102) | 0% (0/102) | 0% (0/102) |
| Normal | 100 % (102/102) | 100 % (102/102) | 100 % (102/102) | 100 % (102/102) | 99 % (101/102) | 100 % (102/102) | 98 % (100/102) | 100 % (102/102) |
| Fat | 0% (0/102) | 0% (0/102) | 0% (0/102) | 0% (0/102) | 1 % (1/102) | 0% (0/102) | 2 % (2/102) | 0% (0/102) |
| Pale (yes/no) | 0 % (0/101) | 0 % (0/102) | 0% (0/100) | 0 % (0/102) | 0 % (0/102) | 0 % (0/102) | 0 % (0/102) | 0 % (0/102) |
| Long hair (yes/no) | 0 % (0/101) | 1% (1/102) | 0 % (0/102) | 0 % (0/102) | 0 % (0/102) | 0 % (0/102) | 0 % (0/102) | 0 % (0/102) |
| Restricted growth (yes/no) | 0% (0/101) | 0 % (0/102) | 0 % (0/102) | 0 % (0/102) | 1 % (1/102) | 0 % (0/102) | 0 % (0/102) | 0 % (0/102) |
| Respiration (normal/abnormal) | 0 % (0/100) | 0 % (0/102) | 1 % (1/102) | 0 % (0/102) | 3 % (3/102) | 0 % (0/102) | 1 % (1/99) | 1 % (1/102) |
| Nasal discharge (yes/no) | 0 % (0/100) | 0 % (0/102) | 1 % (1/102) | 0 % (0/102) | 0 % (0/102) | 0 % (0/102) | 0 % (0/99) | 0 % (0/102) |
| Rear part soiled (diarrhoea) (yes/no) | 5 % (5/100) | 9 % (9/102) | 4 % (4/102) | 1 % (1/102) | 4 % (4/102) | 4 % (4/102) | 6 % (6/99) | 8 % (8/102) |
| Ear wound(s) (yes/no) | 8 % (8/100) | 3 % (3/102) | 19 % (19/102) | 1 % (1/102) | 4 % (4/101) | 0 % (0/102) | 12 % (12/99) | 3 % (3/102) |
| Othematoma (acute or chronic) (yes/no) | 0 % (0/100) | 0 % (0/102) | 1 % (1/102) | 0 % (0/102) | 0 % (0/102) | 0 % (0/102) | 0 % (0/99) | 0 % (0/102) |
| Tail wound(s) (yes/no) | 1 % (1/100) | 0 % (0/102) | 2 % (2/102) | 0 % (0/102) | 3 % (3/102) | 0 % (0/102) | 1 % (1/99) | 0 % (0/102) |
| Abscess (yes/no) | 0 % (0/101) | 0 % (0/102) | 0 % (0/101) | 1 % (1/102) | 0 % (0/102) | 0 % (0/102) | 0 % (0/102) | 1 % (1/102) |
| Signs of Pain (yes/no) | 1 % (1/102) | 1 % (1/102) | 4 % (4/99) | 5 % (5/102) | 1 % (1/98) | 0 % (0/102) | 3 % (3/102) | 1 % (1/102) |
