# Supplementary material for: Comparing video examinations with physical clinical examinations using finishing pigs with umbilical outpouchings as a model
Source: Acta Vet Scand. 2023 Jun 24;65:26. doi: 10.1186/s13028-023-00689-8 (PMC10290328; doi:10.1186/s13028-023-00689-8)
Supplement: Supplementary file 3 — Additional file 3: Clinical examination results of umbilical outpouchings in finishing pigs (n = 102 pigs). The umbilical outpouchings were clinically examined during a traditional physical clinical examination in the stable (Physical) and a clinical examination of the same pigs performed by watching recorded video approximately 1 month after the physical examination (Video). The pigs all had umbilical outpouchings and were selected from two herds. Video recording of the individual pigs was made immediately before the physical examination was performed. All pigs were examined both physically and using video by the same four experienced pig veterinarians. [file 13028_2023_689_MOESM3_ESM.docx]

**Additional file 3.** Clinical examination results of umbilical outpouchings in finishing pigs (n=102 pigs). The umbilical outpouchings were clinically examined during a traditional physical clinical examination in the stable (Physical) and a clinical examination of the same pigs performed by watching recorded video approximately one month after the physical examination (Video). The pigs all had umbilical outpouchings and were selected from two herds. Video recording of the individual pigs was made immediately before the physical examination was performed. All pigs were examined both physically and using video by the same four experienced pig veterinarians.

|  | Veterinarian 1 | | Veterinarian 2 | | Veterinarian 3 | | Veterinarian 4 | |
| --- | --- | --- | --- | --- | --- | --- | --- | --- |
| Clinical findings on umbilical outpouching | Physical | Video | Physical | Video | Physical | Video | Physical | Video |
| Shape: Broad | 57 % (57/100) | 69 % (71/102) | 72 % (71/98) | 88 % (90/102) | 64 % (65/102) | 56 % (57/102) | 96 % (98/102) | 98 % (100/102) |
| Globular | 2 % (2/100) | 2 % (2/102) | 0 % (0/98) | 0 % (0/102) | 0 % (0/102) | 1 % (1/102) | 0 % (0/102) | 0 % (0/102) |
| Oblong | 15 % (15/100) | 15 % (15/102) | 19 % (19/98) | 7 % (7/102) | 23 % (23/102) | 33 % (34/102) | 2 % (2/102) | 1 % (1/102) |
| Drop | 26 % (26/100) | 14 % (14/102) | 8 % (8/98) | 5 % (5/102) | 14 % (14/102) | 10 % (10/102) | 2 % (2/102) | 1 % (1/102) |
| Distance from abdominal wall in cm (standard deviation) | 13.7 (4.4) | 10.7 (3.2) | 12.2 (3.5) | 12.6 (2.9) | 1.9 (0.6) | 1.8 (0.6) | 13.0 (3.8) | 12.0 (3.4) |
| Width of the outpouching in cm | 17.9 (4.4) | 17.0 (3.4) | 16.2 (3.4) | 15.9 (3.2) | 17.9 (3.9) | 15.8 (3.4) | 16.2 (3.4) | 15.8 (3.2) |
| Pendulous outpouching (yes/no) | 19 % (19/102) | 23 % (23/102) | 81 % (82/101) | 90 % (92/102) | 79 % (81/102) | 60 % (61/102) | 92 % (94/102) | 100 % (102/102) |
| Firm consistency (yes/no) | 15 % (15/102) | 12 % (12/102) | 7 % (7/102) | 10 % (10/102) | 23 % (23/102) | 5 % (5/102) | 11 % (11/101) | 7 % (7/102) |
| Reducible into the abdomen (yes/no) | 78 % (79/101) | 86 % (88/102) | 85 % (87/102) | 88 % (90/102) | 59 % (61/102) | 90 % (92/102) | 59 % (58/98) | 83 % (85/102) |
| Red skin (yes/no) | 0% (0/102) | 1 % (1/102) | 2 % (2/102) | 0% (0/102) | 7 % (7/102) | 7 % (7/102) | 1 % (1/102) | 0% (0/102) |
| Edema (yes/no) | 0% (0/102) | 0% (0/102) | 1 % (1/102) | 0% (0/102) | 0% (0/102) | 1 % (1/102) | 0% (0/102) | 0% (0/102) |
| Pain induced by palpation of the outpouching: Yes | 0 % (0/102) | 0 % (0/102) | 0 % (0/102) | 0 % (0/102) | 2 % (2/102) | 0 % (0/102) | 0 % (0/102) | 0 % (0/102) |
| No | 100% (102/102) | 100% (102/102) | 100% (102/102) | 100% (102/102) | 98 % (100/102) | 100% (102/102) | 100% (102/102) | 98 % (100/102) |
| Don't know | 0 % (0/102) | 0 % (0/102) | 0 % (0/102) | 0 % (0/102) | 0 % (0/102) | 0 % (0/102) | 0 % (0/102) | 2 % (2/102) |
| Touching the floor (yes/no) | 0 % (0/102) | 0 % (0/102) | 2 % (2/101) | 1 % (1/102) | 0 % (0/102) | 0 % (0/102) | 0 % (0/100) | 0 % (0/102) |
| Distance to floor in cm (standard deviation) | 15.0 (4.3) | 15.4 (3.4) | 13.0 (4.2) | 14.5 (3.5) | 13.8 (4.1) | 15.2 (3.4) | 14.3 (4.1) | 14.8 (3.6) |
| Touching the legs (yes/no) | 4 % (4/102) | 2 % (2/102) | 10 % (10/101) | 3 % (3/102) | 1 % (1/102) | 0 % (0/102) | 8 % (8/102) | 1 % (1/101) |
| Restricted movements caused by outpouching (yes/no) | 0 % (0/102) | 0 % (0/102) | 0 % (0/102) | 0 % (0/102) | 2 % (2/102) | 3 % (3/102) | 0 % (0/101) | 0 % (0/102) |
| Vascularization (macroscopic) (yes/no) | 1 % (1/102) | 1 % (1/102) | 15 % (15/101) | 5 % (5/102) | 5 % (5/102) | 2 % (2/102) | 19 % (19/99) | 16 % (16/102) |
| Free movable skin: Yes | 84 % (84/100) | 89 % (91/102) | 96 % (97/101) | 95 % (97/102) | 75 % (77/102) | 56 % (57/102) | 97 % (99/102) | 96 % (98/102) |
| No | 16 % (16/100) | 11 % (11/102) | 4 % (4/101) | 5 % (5/102) | 24 % (24/102) | 44 % (45/102) | 3 % (3/102) | 4 % (4/102) |
| Don't know | 0 % (0/100) | 0 % (0/102) | 0% (0/101) | 0 % (0/102) | 1 % (1/102) | 0 % (0/102) | 0 % (0/102) | 0 % (0/102) |
| Scar(s) (yes/no) | 33 % (34/102) | 32 % (33/102) | 38 % (39/102) | 70% (71/102) | 6 % (6/102) | 1 % (1/102) | 12 % (12/101) | 14 % (14/102) |
| Excoriation(s) (yes/no) | 16 % (16/100) | 6 % (6/102) | 20 % (20/102) | 25 % (26/102) | 47 % (48/102) | 29 % (30/102) | 18 % (18/101) | 25 % (25/102) |
| Contusion(s) (yes/no) | 1 % (1/102) | 0 % (0/102) | 0 % (0/102) | 7 % (7/102) | 0 % (0/102) | 0 % (0/102) | 0 % (0/101) | 3 % (3/102) |
| Wound(s) (yes/no) | 41 % (42/102) | 47 % (48/102) | 35 % (35/101) | 48 % (49/102) | 42 % (43/102) | 48 % (49/102) | 47 % (47/101) | 48 % (49/102) |
| Number of wounds > 4 cm^2^: 0 | 49 % (20/41) | 44 % (21/48) | 45 % (15/33) | 49 % (24/49) | 37 % (16/43) | 49 % (24/49) | 51 % (23/45) | 47 % (23/49) |
| 1 | 51 % (21/41) | 56 % (27/48) | 52 % (17/33) | 51 % (25/49) | 60% (26/43) | 51 % (25/49) | 49 % (22/45) | 53 % (26/49) |
| ≥2 | 0 % (0/41) | 0 % (0/48) | 3 % (1/33) | 0 % (0/49) | 2 % (1/43) | 0 % (0/49) | 0 % (0/45) | 0 % (0/49) |
