# Supplementary material for: Comparing video examinations with physical clinical examinations using finishing pigs with umbilical outpouchings as a model
Source: Acta Vet Scand. 2023 Jun 24;65:26. doi: 10.1186/s13028-023-00689-8 (PMC10290328; doi:10.1186/s13028-023-00689-8)
Supplement: Supplementary file 4 — Additional file 4: Clinical examination results of wounds (n = 49 wounds) located on umbilical outpouchings in 49 different finishing pigs. The wounds were clinically examined during a traditional physical clinical examination in the stable (Physical) and a clinical examination of the same wounds performed by watching recorded video approximately 1 month after the physical examination (Video). The pigs all had umbilical outpouchings and were selected from two herds. Video recording of the individual pigs was made immediately before the physical examination was performed. All pigs were examined both physically and using video by the same four experienced pig veterinarians. [file 13028_2023_689_MOESM4_ESM.docx]

**Additional file 4.** Clinical examination results of wounds (n=49 wounds) located on umbilical outpouchings in 49 different finishing pigs. The wounds were clinically examined during a traditional physical clinical examination in the stable (Physical) and a clinical examination of the same wounds performed by watching recorded video approximately one month after the physical examination (Video). The pigs all had umbilical outpouchings and were selected from two herds. Video recording of the individual pigs was made immediately before the physical examination was performed. All pigs were examined both physically and using video by the same four experienced pig veterinarians.

|  | | | |  |  |  |  |  |
| --- | --- | --- | --- | --- | --- | --- | --- | --- |
|  | Veterinarian 1 | | Veterinarian 2 | | Veterinarian 3 | | Veterinarian 4 | |
| Clinical findings on wounds | Physical | Video | Physical | Video | Physical | Video | Physical | Video |
|  |  |  |  |  |  |  |  |  |
| Wound type: Vulnus | 96 % (44/46) | 82 % (40/49) | 100 % (45/45) | 100 % (49/49) | 92 % (44/48) | 84 % (41/49) | 50 % (24/48) | 88 % (43/49) |
| Ulcus | 4 % (2/46) | 18 % (9/49) | 0 % (0/45) | 0 % (0/49) | 8 % (4/48) | 16 % (8/49) | 50 % (24/48) | 12 % (6/49) |
| Wound length in cm (standard deviation) | 2.5 (1.8) | 2.6 (1.8) | 2.7 (1.9) | 2.7 (2.1) | 3.0 (2.1) | 2.8 (2.1) | 2.6 (1.7) | 2.6 (1.9) |
| Wound width in cm (standard deviation) | 1.8 (1.7) | 2.2 (2.0) | 1.8 (1.1) | 2.0 (1.3) | 1.9 (1.0) | 1.9 (1.1) | 2.0 (1.6) | 2.2 (1.6) |
| Length * width (standard deviation) | 6.6 (9.0) | 7.6 (11.8) | 6.2 (7.4) | 6.9 (8.8) | 6.9 (7.6) | 6.7 (9.0) | 7.2 (9.0) | 7.9 (11.2) |
| Length + width (standard deviation) | 4.4 (3.2) | 4.8 (3.3) | 4.5 (2.8) | 4.7 (3.0) | 4.9 (2.8) | 4.6 (3.0) | 4.7 (3.1) | 4.8 (3.2) |
| Location: Side | 10 % (5/49) | 2 % (1/49) | 9 % (4/47) | 14 % (7/49) | 6 % (3/49) | 6 % (3/49) | 19% (9/47) | 8 % (4/49) |
| Bottom | 90 % (44/49) | 98 % (48/49) | 91 % (43/47) | 86 % (42/49) | 94 % (46/49) | 94 % (46/49) | 81 % (38/47) | 92 % (45/49) |
| Crust on wound: No | 86 % (42/49) | 74 % (36/49) | 31 % (15/49) | 22 % (11/49) | 20% (10/49) | 39 % (19/49) | 86 % (42/49) | 76 % (37/49) |
| Partial | 10 % (5/49) | 24 % (12/49) | 65 % (32/49) | 78 % (38/49) | 76 % (37/49) | 59 % (29/49) | 12 % (6/49) | 22 % (11/49) |
| Yes | 4 % (2/49) | 2 % (1/49) | 4 % (2/49) | 0 % (0/49) | 4 % (2/49) | 2 % (1/49) | 2 % (1/49) | 2 % (1/49) |
| Redness around wound (yes/no) | 0 % (0/49) | 0 % (0/49) | 12 % (6/49) | 16 % (8/49) | 2 % (1/49) | 18 % (9/49) | 2 % (1/49) | 0 % (0/49) |
| Wound pus (yes/no) | 0 % (0/49) | 4 % (2/49) | 0 % (0/49) | 4 % (2/49) | 0 % (0/49) | 8 % (4/49) | 0 % (0/49) | 2 % (1/49) |
| Active bleeding from wound (yes/no) | 6 % (3/49) | 10 % (5/49) | 10 % (5/49) | 20 % (10/49) | 2 % (1/49) | 6 % (3/49) | 4 % (2/49) | 10 % (5/49) |
| Wound suppuration (yes/no) | 6 % (3/49) | 59 % (26/49) | 20 % (10/49) | 94 % (46/49) | 0 % (0/49) | 29 % (14/49) | 0 % (0/49) | 0 % (0/49) |
| Wound pain: Yes | 0 % (0/49) | 0 % (0/49) | 0 % (0/49) | 0 % (0/49) | 0 % (0/49) | 0 % (0/49) | 0 % (0/49) | 0 % (0/49) |
| No | 100 % (49/49) | 100 % (49/49) | 100 % (49/49) | 100 % (49/49) | 88 % (43/49) | 96 % (47/49) | 100 % (49/49) | 90 % (44/49) |
| Don't know | 0 % (0/49) | 0 % (0/49) | 0 % (0/49) | 0 % (0/49) | 12 % (6/49) | 4 % (2/49) | 0 % (0/49) | 10 % (5/49) |
| Necrosis in wound (yes/no) | 8 % (4/49) | 10 % (5/49) | 0 % (0/49) | 0 % (0/49) | 59 % (29/49) | 43 % (21/49) | 6 % (3/49) | 4 % (2/49) |
| Fistula in wound (yes/no) | 4 % (2/49) | 2 % (1/49) | 2 % (1/49) | 2 % (1/49) | 0 % (0/49) | 2 % (1/49) | 2 % (1/49) | 4 % (2/49) |
| Presence of granulation tissue (yes/no) | 43 % (21/49) | 16 % (8/49) | 94 % (46/49) | 96 % (47/49) | 78 % (38/49) | 82 % (40/49) | 94 % (46/49) | 100 % (49/49) |
| Re-epithelization (yes/no) | 98 % (47/48) | 90 % (44/49) | 90 % (44/49) | 76 % (37/49) | 100 % (49/49) | 90 % (44/49) | 98 % (48/49) | 100 % (49/49) |
| Thick wound edges (yes/no) | 92 % (44/48) | 35 % (17/49) | 92 % (45/49) | 84 % (41/49) | 53 % (26/49) | 41 % (20/49) | 65 % (32/49) | 67 % (33/49) |
| Connective tissue (yes/no) | 55 % (27/49) | 8 % (4/49) | 24 % (12/49) | 2 % (1/49) | 6 % (3/49) | 8 % (4/49) | 52 % (25/48) | 53 % (26/49) |
| Progression of healing: Partially healed | 17 % (8/46) | 4 % (2/49) | 76 % (37/49) | 98 % (48/49) | 86 % (42/49) | 67 % (33/49) | 4 % (2/49) | 2 % (1/49) |
| Not healed | 83 % (38/46) | 96 % (47/49) | 24 % (12/49) | 2 % (1/49) | 14 % (7/49) | 33 % (16/49) | 96 % (47/49) | 98 % (48/49) |
| Severe wound (yes/no) | 43 % (21/49) | 43 % (21/49) | 22 % (11/49) | 35 % (17/49) | 22 % (11/49) | 37 % (18/49) | 14 % (7/49) | 27 % (13/49) |
| Open wound (yes/no) | 88 % (43/49) | 98 % (48/49) | 55 % (27/49) | 41 % (20/49) | 88 % (43/49) | 98 % (48/49) | 98 % (48/49) | 98 % (48/49) |
| Prognosis for total healing: Good | 82 % (40/49) | 71 % (35/49) | 96 % (47/49) | 71 % (35/49) | 69 % (34/49) | 63 % (31/49) | 90 % (44/49) | 65 % (32/49) |
| Guarded | 16 % (8/49) | 24 % (12/49) | 4 % (2/49) | 29 % (14/49) | 27 % (13/49) | 33 % (16/49) | 10 % (5/49) | 29 % (14/49) |
| Poor | 2 % (1/49) | 4 % (2/49) | 0 % (0/49) | 0% (0/49) | 4 % (2/49) | 4 % (2/49) | 0 % (0/49) | 6 % (3/49) |
