# Supplementary material for: Comparing video examinations with physical clinical examinations using finishing pigs with umbilical outpouchings as a model
Source: Acta Vet Scand. 2023 Jun 24;65:26. doi: 10.1186/s13028-023-00689-8 (PMC10290328; doi:10.1186/s13028-023-00689-8)
Supplement: Supplementary file 5 — Additional file 5: Fitness for transport evaluation of 102 finishing pigs performed by four experienced pig veterinarians. All pigs were clinically examined during a traditional physical clinical examination in the stable (Physical) and a clinical examination of the same pigs was performed by watching recorded video approximately 1 month after the physical examination (Video). The pigs all had umbilical outpouchings and were selected from two herds. Video recording of the individual pigs was made immediately before the physical examination was performed. All pigs were examined both physically and using video by the same four experienced pig veterinarians. [file 13028_2023_689_MOESM5_ESM.docx]

**Additional file 5.** Fitness for transport evaluation of 102 finishing pigs performed by four experienced pig veterinarians. All pigs were clinically examined during a traditional physical clinical examination in the stable (Physical) and a clinical examination of the same pigs was performed by watching recorded video approximately one month after the physical examination (Video). The pigs all had umbilical outpouchings and were selected from two herds. Video recording of the individual pigs was made immediately before the physical examination was performed. All pigs were examined both physically and using video by the same four experienced pig veterinarians.

|  | | |  |  |  |  |  |  |
| --- | --- | --- | --- | --- | --- | --- | --- | --- |
|  | Veterinarian 1 | | Veterinarian 2 | | Veterinarian 3 | | Veterinarian 4 | |
|  | Physical | Video | Physical | Video | Physical | Video | Physical | Video |
|  |  |  |  |  |  |  |  |  |
| Health status: |  |  |  |  |  |  |  |  |
| General good health (score 1) | 84 % (86/102) | 98 % (100/102) | 99 % (99/100) | 97 % (99/102) | 98 % (100/102) | 99 % (101/102) | 93 % (94/101) | 69 % (70/102) |
| Minor affected health (score 2) | 13 % (13/102) | 0 % (0/102) | 0 % (0/100) | 1 % (1/102) | 1 % (1/102) | 1 % (1/102) | 2 % (2/101) | 26 % (27/102) |
| Affected health (score 3) | 3 % (3/102) | 2 % (2/102) | 1 % (1/100) | 2 % (2/102) | 1 % (1/102) | 0 % (0/102) | 5 % (5/101) | 5 % (5/102) |
|  |  |  |  |  |  |  |  |  |
| Likelyhood for health deterioration during transport  when the pig is separated from other pigs: |  |  |  |  |  |  |  |  |
| Small | 70 % (71/102) | 87 % (89/102) | 93 % (95/102) | 99 % (101/102) | 96 % (98/102) | 91 % (93/102) | 91 % (93/102) | 78 % (80/102) |
| Medium | 28 % (29/102) | 13 % (13/102) | 6 % (6/102) | 1 % (1/102) | 4 % (4/102) | 9 % (9/102) | 4 % (4/102) | 20 % (20/102) |
| High | 2 % (2/102) | 0 % (0/102) | 1 % (1/102) | 0 % (0/102) | 0 % (0/102) | 0 % (0/102) | 5 % (5/102) | 2 % (2/102) |
|  |  |  |  |  |  |  |  |  |
| Likelyhood for health deterioration during transport  when the pig is not separated from other pigs: | |  |  |  |  |  |  |  |
| Small | 31 % (32/102) | 47 % (48/102) | 87 % (89/102) | 42 % (43/102) | 95 % (97/102) | 91 % (93/102) | 68 % (69/102) | 34 % (35/102) |
| Medium | 58 % (59/102) | 48 % (49/102) | 8 % (8/102) | 39 % (40/102) | 5 % (5/102) | 9 % (9/102) | 23 % (23/102) | 45 % (46/102) |
| High | 11 % (11/102) | 5 % (5/102) | 5 % (5/102) | 19 % (19/102) | 0 % (0/102) | 0 % (0/102) | 10 % (10/102) | 21 % (21/102) |
|  |  |  |  |  |  |  |  |  |
| Fit for transport (normal conditions) (yes/no) | 12 % (12/102) | 15 % (15/102) | 32 % (33/102) | 16 % (16/102) | 24 % (24/102) | 34 % (35/102) | 10 % (10/102) | 10 % (10/102) |
| Fit for transport with extra consideration of comfort (yes/no) | 53 % (54/102) | 37 % (38/102) | 52 % (53/102) | 64 % (65/102) | 51 % (52/102) | 35 % (36/102) | 67 % (68/102) | 63 % (64/102) |
| Not fit for transport (yes/no) | 35 % (36/102) | 48 % (49/102) | 16 % (16/102) | 21 % (21/102) | 25 % (26/102) | 30 % (31/102) | 24 % (24/102) | 27 % (28/102) |
